# Non-pharmacological and physical strategies for managing dental anxiety: a systematic review with implications for dental education

**DOI:** 10.3389/phrs.2026.1609316

**Published:** 2026-07-31

**Authors:** Angelika Wawrzyniak, Natalia Sadowska, Maria Pawlak-Mojsiewicz, Natalia Walczuk, Diana Masłyk, Izabela Barczyk, Piotr Skomro, Magdalena Sroczyk-Jaszczyńska, Karina Kijak, Helena Gronwald, Lidia Szczucka, Adam Andrzej Garstka, Danuta Lietz-Kijak

**Affiliations:** 1 Research Club STO-MATER-FIZ, Department of Propaedeutics, Physical Diagnostics and Dental Physiotherapy, Pomeranian Medical University in Szczecin, Szczecin, Poland; 2 Department of Propaedeutics, Physical Diagnostics and Dental Physiotherapy, Faculty of Medicine and Dentistry, Pomeranian Medical University in Szczecin, Szczecin, Poland; 3 Department of General, Dental and Interventional Radiology, Faculty of Medicine and Dentistry, Pomeranian Medical University, Szczecin, Poland

**Keywords:** behavioral management, dental anxiety, dentophobia, non-pharmacological interventions, patient cooperation

## Abstract

**Objectives:**

Dental anxiety remains a significant barrier to effective oral healthcare, contributing to treatment avoidance and poorer clinical outcomes. This systematic review aimed to evaluate the effectiveness of non-pharmacological and physical interventions in reducing dental anxiety and improving patient cooperation.

**Methods:**

A systematic search was conducted in PubMed/MEDLINE, Scopus, Dentistry & Oral Sciences Source, and Google Scholar for studies published between 1971 and 2024. Randomized controlled trials, quasi-experimental, and observational studies assessing non-pharmacological or technology-assisted interventions in children and adults were included. Study selection and data extraction were performed independently by two reviewers. The certainty of evidence was assessed using the GRADE framework.

**Results:**

A total of 126 studies were included in the qualitative synthesis. Interventions were grouped into three categories: technological/physical (e.g., computer-controlled anesthesia, Er:YAG laser, TENS), sensory-relaxation (e.g., aromatherapy, breathing techniques, hypnosis), and digital/interactive (e.g., mobile applications, audiovisual distraction, humanoid robots). The majority of studies reported reductions in anxiety or improvements in patient cooperation. The strongest evidence supported computer-controlled anesthesia systems while moderate-certainty evidence was identified for aromatherapy, laser-based interventions, and audiovisual distraction. Several additional interventions demonstrated potentially beneficial effects, although the certainty of evidence remained limited due to methodological weaknesses.

**Conclusion:**

Non-pharmacological and physical interventions can effectively reduce dental anxiety and enhance treatment acceptance across diverse patient populations. Integrating these strategies into routine clinical practice and dental education may improve patient-centered care and reduce reliance on pharmacological methods. Further high-quality studies are needed to confirm long-term effectiveness and support implementation.

**Systematic Review Registration:**

https://www.crd.york.ac.uk/PROSPERO/view/CRD420251229878, identifier CRD420251229878.

## Introduction

Dental anxiety remains a common and clinically significant problem affecting both children and adults undergoing dental treatment. It is considered a multifactorial condition associated with fear, stress, avoidance of dental care, and poorer oral health outcomes [1–5]. High levels of dental anxiety may negatively influence patient cooperation, increase treatment difficulty, prolong clinical procedures, and contribute to delayed or irregular dental attendance [1, 5, 6]. In pediatric patients, negative early dental experiences may additionally influence future attitudes toward oral healthcare and treatment acceptance [7, 8]. The etiology of dental anxiety is complex and may involve previous traumatic experiences, fear of pain, environmental stimuli, loss of control, and individual psychological characteristics [1–3]. Fear associated with local anesthesia and invasive dental procedures remains one of the most commonly reported triggers of anxiety in dental settings [9, 10]. Several contemporary anxiety-management approaches are based on behavioral conditioning and neurophysiological mechanisms, including the gate control theory of pain and related concepts of pain modulation [11, 12]. These concepts have contributed to the development of interventions aimed at modifying pain perception, emotional response, and patient attention during dental treatment. Traditionally, management of dental anxiety has relied on communication techniques, behavioral management strategies, conscious sedation, and pharmacological interventions [9, 13]. However, growing interest has recently focused on non-pharmacological and minimally invasive methods that may improve patient comfort while reducing the need for pharmacological sedation in selected patients [1, 14]. Technological advances have introduced computer-controlled local anesthetic delivery systems, needle-free anesthesia, laser-assisted procedures, virtual reality distraction, mobile applications, and sensory-adapted environments as potential adjunctive tools in anxiety management [15–32]. A growing number of studies have also investigated complementary and sensory-based interventions such as aromatherapy, breathing exercises, hypnosis, audiovisual distraction, transcutaneous electrical nerve stimulation (TENS), and animal-assisted therapy [32–50], [51–75]. Several of these approaches demonstrated beneficial effects in reducing anxiety, pain perception, and behavioral distress during dental treatment, particularly among pediatric and special care patients [27, 37, 38, 53, 66, 68, 76]. Environmental and interpersonal factors may additionally influence patient cooperation and emotional responses during dental treatment [77–81]. Studies evaluating sensory-adapted dental environments and waiting room modifications have suggested that relatively simple environmental interventions may improve patient comfort and reduce stress associated with dental visits [82–88]. Despite the increasing number of available interventions, the current evidence remains heterogeneous with respect to study design, intervention protocols, outcome assessment methods, and methodological quality. Previous reviews have evaluated selected anxiety-reduction strategies in medical and dental settings. However, comprehensive synthesis of non-pharmacological and technology-assisted interventions used specifically in contemporary dental practice remains limited [4, 45, 63, 89, 90]. In addition, newer technologies and patient-centered approaches introduced in recent years were not fully represented in earlier reviews. Therefore, the aim of this systematic review was to evaluate the effectiveness of contemporary non-pharmacological and technology-assisted interventions used to reduce dental anxiety in children and adults undergoing dental treatment. The review also aimed to assess the certainty of evidence using the GRADE approach and discuss the potential clinical and educational implications of these interventions in modern dental practice.

### Aim of the study

The primary aim of this systematic review was to evaluate the effectiveness of non-pharmacological and physical interventions in reducing dental anxiety and dentophobia among children and adults undergoing dental treatment. Specifically, the review sought to:

Identify and classify available unconventional and physical methods used in clinical and public health dentistry to reduce anxiety.

Determine which interventions demonstrate the strongest evidence of effectiveness in improving patient comfort, cooperation, and treatment acceptance.

Compare these approaches with conventional anxiety-management techniques; and Assess the overall certainty and quality of evidence using the GRADE framework.

This review was conducted in accordance with the PRISMA 2020 guidelines and was prospectively registered in the PROSPERO database (CRD420251229878).

## Methods

### Study design and registration

This systematic review was conducted in accordance with the PRISMA 2020 guidelines and was prospectively registered in the PROSPERO database (CRD420251229878). The review followed a predefined protocol based on PICOS criteria (Population, Intervention, Comparator, Outcome, Study Design).

### Eligibility criteria

Studies were included if they met the following criteria:

Population: children, adolescents, or adults reporting dental anxiety or dentophobia;

Intervention: non-pharmacological, physical, sensory, or technology-assisted strategies implemented in a dental setting.

Comparator: conventional anxiety-management methods, placebo, or no intervention;

Outcomes: changes in anxiety level, treatment cooperation, pain perception, physiological stress markers (e.g., heart rate, blood pressure, salivary cortisol);

Study type: randomized controlled trials, quasi-experimental studies, and observational studies;

Language: English, Polish, or German; Publication date: 1971–2024.

Exclusion criteria included animal studies, *in vitro* or simulation-based studies, dental interventions unrelated to anxiety, and narrative reviews or opinion pieces without original data.

### Information sources and search strategy

A comprehensive and systematic literature search was conducted in the following electronic databases: PubMed/MEDLINE, Scopus, Dentistry & Oral Sciences Source (EBSCO), and Google Scholar. The search covered studies published from January 1971 to December 2024. The search strategy combined controlled vocabulary (e.g., MeSH terms) and free-text keywords related to dental anxiety and non-pharmacological interventions. Boolean operators (“AND”, “OR”) and truncation were applied as appropriate. The search strategy was adapted for each database to maximize sensitivity and specificity of retrieved records. Detailed search strategies for each database are provided in Supplementary Material 1. In addition, the reference lists of all included studies and relevant reviews were manually screened to identify further eligible publications. Duplicate records were removed using Mendeley reference management software. The inclusion of studies published between 1971 and 2024 was intentional to capture the full historical development of non-pharmacological approaches to dental anxiety management. Early studies from the 1970s and 1980s established fundamental psychological and neurophysiological concepts, including behavioral conditioning, relaxation techniques, hypnosis, and the gate control theory of pain, which underpin many contemporary interventions. Over subsequent decades, these foundational approaches evolved and were complemented by technological advancements, such as computer-controlled local anesthesia systems, laser-assisted dentistry, and digital or interactive tools including virtual reality and mobile applications. Including studies across this extended time frame enabled a comprehensive synthesis of both classical and modern strategies, allowing for a better understanding of how current interventions have emerged and how their mechanisms are conceptually linked to earlier evidence. Furthermore, given the heterogeneity of interventions and the relatively recent emergence of some technologies, restricting the analysis to more recent studies alone could have excluded clinically relevant foundational evidence. Therefore, the broad time range enhances the conceptual completeness of the review while maintaining clinical relevance through critical appraisal and GRADE-based evaluation of evidence quality.

### Study selection

**FIGURE 1:**
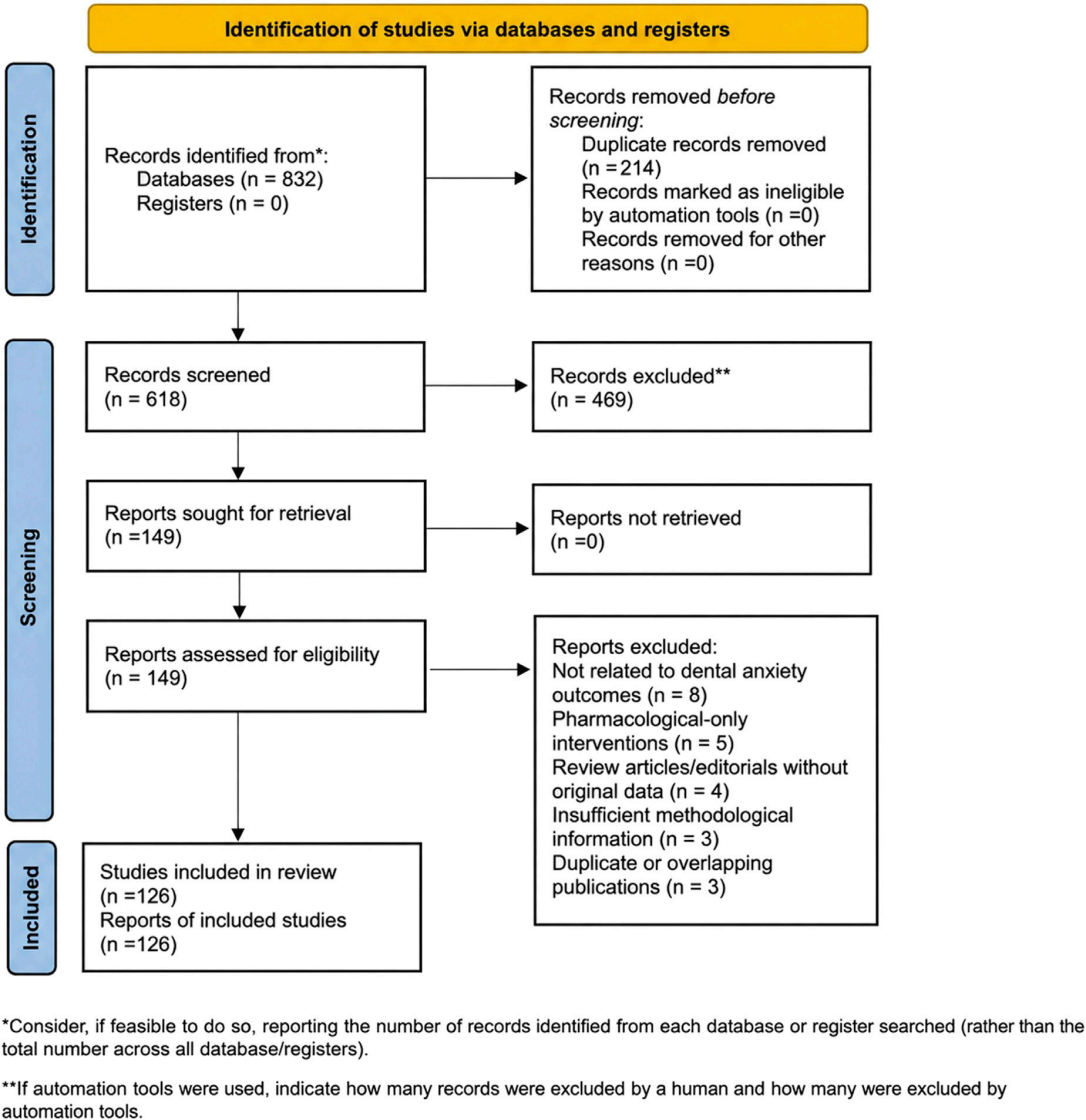
PRISMA 2020 flow diagram showing the identification, screening, eligibility assessment, and inclusion of studies examining non-pharmacological and physical interventions for managing dental anxiety in children and adults (systematic review, global studies, 1971–2024).

Two reviewers independently screened titles and abstracts identified through the database search. Full texts of potentially relevant articles were subsequently assessed according to the predefined eligibility criteria. Any disagreements between reviewers were resolved through discussion until consensus was achieved. During the eligibility assessment, records were excluded because of lack of relevance to dental anxiety management, exclusively pharmacological interventions, absence of original research data, conference abstracts without full-text availability, duplicate publications, insufficient methodological information, or study designs not meeting the predefined inclusion criteria. Primary empirical studies were included in the qualitative synthesis, while selected reviews and theoretical publications were used as background references in the Introduction and Discussion. Table 1 presents representative examples of included studies, including publication year, country, study design, study population, intervention type, comparator, and primary outcomes. The complete study selection process, including identification, screening, eligibility assessment, and reasons for exclusion, is summarized in the PRISMA 2020 flow diagram (Figure 1).

**TABLE 1:** Representative examples of included studies, including publication year, country, study design, study population, intervention type, comparator, and primary outcomes (systematic review, global studies, 1971–2024).

Author (Year)	Country	Study design	Population	Intervention	Comparator	Outcome measures	Main findings
Ghaderi et al. [30]	Iran	Randomized controlled trial	Children (n = 60)	Lavender aromatherapy	No aromatherapy	Stress level, salivary cortisol	Significant reduction in stress levels and anxiety
Elicherla et al. [26]	India	Randomized controlled trial	Children (n = 50)	Mobile application (“little lovely dentist”)	Tell-show-do technique	Anxiety scale scores	Mobile application reduced anxiety more effectively than conventional behavioral guidance
Shankar et al. [16]	India	Clinical comparative study	Adults with periodontitis (n = 30)	Needle-free jet anesthesia	Conventional syringe anesthesia	Pain and anxiety scores	Reduced pain perception and dental anxiety
Rizzo-Lorenzo et al. [91]	Spain	Randomized clinical study	Adults (n = 40)	Pre-procedural explanation of computer-controlled anesthesia	Standard information	Anxiety levels	Detailed procedural information increased anticipatory anxiety in some patients
Kasimoglu et al. [92]	Turkey	Randomized controlled trial	Children (n = 60)	Humanoid robot assistance (iRobiQ)	Conventional dental visit	Heart rate, anxiety scores	Reduced anxiety and improved cooperation
Zink et al. [25]	Brazil	Observational clinical study	Children with ASD (n = 20)	Communication support application	Standard communication	Behavior and cooperation	Improved communication and reduced treatment-related stress
Bahrololoomi et al. [42]	Iran	Randomized controlled trial	Children (n = 60)	Bubble breathing exercise	No breathing intervention	Pain and anxiety during injection	Significant reduction in pain and anxiety
Sayed et al. [93]	India	Clinical comparative study	Children (n = 40)	Live video visualization using dental operating microscope	Conventional treatment	Anxiety scores	Reduced anxiety during restorative procedures
Abdrabuh et al. [94]	Saudi Arabia	Randomized split-mouth clinical study	Children (n = 35)	Er:YAG laser therapy	Conventional rotary instruments	Anxiety and pain perception	Lower anxiety levels and reduced pain perception with laser treatment
Shetty et al. [95]	India	Randomized controlled trial	Children (n = 50)	Audiovisual distraction using virtual reality	Standard care	Venham anxiety scale	Significant reduction in anxiety during treatment
Fux-Noy et al. [29]	Israel	Randomized controlled trial	Children	Computer-controlled local anesthetic delivery (CCLAD)	Conventional syringe injection	Pain and anxiety scores	Reduced injection-related anxiety and pain
Glaesmer et al. [96]	Germany	Controlled clinical trial	Adults undergoing tooth extraction (n = 102)	Hypnosis adjunctive therapy	Treatment as usual	Dental anxiety before, during, and after treatment	Hypnosis reduced anxiety during tooth removal and was well accepted by patients

### Data extraction

Data were extracted independently by two reviewers using a standardized form. Extracted variables included authorship, year and country, study design, sample size, type of intervention, comparator, anxiety measurement tool, and reported outcomes. Discrepancies were resolved through consensus.

### Risk of bias assessment

The methodological quality and risk of bias of the included studies were independently evaluated by two reviewers using validated assessment tools appropriate to the study design. Randomized controlled trials were assessed using the Cochrane Risk of Bias 2 (RoB 2) tool, whereas non-randomized and observational studies were evaluated using Joanna Briggs Institute (JBI) critical appraisal tools. The assessment considered domains including selection bias, allocation concealment, blinding, incomplete outcome data, selective reporting, and methodological consistency. Disagreements between reviewers were resolved through discussion and consensus. The results of the risk of bias assessment informed the GRADE certainty ratings presented in Table 3.

### Justification of GRADE ratings

High-certainty evidence for computer-controlled anesthesia was supported by multiple well-designed randomized controlled trials demonstrating consistent reductions in pain and anxiety outcomes. Moderate-certainty evidence for aromatherapy and laser-based interventions was due to generally positive findings but with variability in protocols and outcome measures. Interventions such as TENS, hypnosis, and digital technologies were downgraded due to methodological limitations including small sample sizes, lack of blinding, and heterogeneity in anxiety assessment tools. In several cases, indirectness was noted due to differences in patient populations (e.g., children vs. adults) and clinical settings. Imprecision was frequently present due to wide confidence intervals or limited statistical power.

### Data synthesis

Due to heterogeneity in interventions, populations, and outcome measures, a narrative synthesis was performed. Results were grouped by intervention type and patient population. Meta-analysis was not attempted because of substantial methodological variability across studies.

## Results

### Study selection

A total of 832 records were identified through database searching. After removal of 214 duplicate records, 618 records were screened by title and abstract, and 469 records were excluded. The majority of records excluded during title and abstract screening were unrelated to dental anxiety management, focused exclusively on pharmacological interventions, or did not include original clinical data. Subsequently, 149 full-text articles were assessed for eligibility. Of these, 23 studies were excluded because they did not meet the predefined inclusion criteria, lacked sufficient methodological information, or did not report relevant anxiety-related outcomes. Finally, 126 studies met all eligibility criteria and were included in the qualitative synthesis. The study selection process is presented in the PRISMA 2020 flow diagram (Figure 1). Table 1 summarizes representative examples of included studies. Detailed characteristics of included studies and selected background references are presented in Supplementary Material 2.

### Characteristics of included studies

The included studies were published between 1971 and 2024 and represented diverse geographic regions and clinical settings. The reviewed literature included studies involving adult, pediatric, and mixed patient populations. The included literature primarily consisted of randomized controlled trials (n = 63), quasi-experimental studies (n = 41), and observational studies (n = 22). Table 1 summarizes representative examples of included studies, including publication year, study design, patient population, intervention type, comparator, and outcome measures.

### Categorization of the interventions

The included interventions were grouped into three main categories: technological and physical approaches, sensory and relaxation-based techniques, and digital or interactive interventions, as summarized in Table 2.

**TABLE 2:** Categorization of non-pharmacological and physical interventions used to reduce dental anxiety, grouped by mechanism of action, target population, and clinical applicability (systematic review, global studies, 1971–2024).

Intervention	Mechanism of action	Certainty of evidence (GRADE)	Target population	Advantages	Limitations	Reported patient acceptance
Aromatherapy	Olfactory stimulation influencing the limbic system	Moderate	Children and adults	Non-invasive, pleasant, improves mood	Effect varies by scent; limited standardization	High
Mobile applications	Cognitive distraction, education, desensitization	Low	Children (especially ASD)	Interactive, engaging, accessible at home	Limited evidence; mostly non-invasive procedures	High
TENS	Neuromodulation and endorphin release (gate control mechanism)	Low	Children	Non-invasive, adjustable intensity	Limited to mild pain; limited evidence	Moderate
Computer-controlled anesthesia (CCLAD/STA)	Controlled anesthetic delivery, reduced injection pain	High	Children and adults	Reduced pain, consistent delivery, increased control	Higher cost; requires training	High
Needle-free anesthesia	Jet injection without needle	Moderate	Children and adults	Avoids needle-related fear; rapid onset	Pressure discomfort; limited indications	Variable
Breathing exercises	Parasympathetic activation, stress reduction	Moderate	Children and adults	Low cost, easy to implement	Requires patient cooperation	High
Dental operating microscope (DOM)	Visual distraction and enhanced procedural control	Low	Children	Dual clinical and psychological benefit	High cost; limited applicability	Moderate
Humanoid robots	Multisensory distraction and emotional engagement	Low	Children	Improves cooperation and reduces fear	High cost; limited availability	High
Hypnosis	Cognitive modulation and relaxation through suggestion	Low	Adults	Non-invasive; potentially strong effect	Requires trained personnel; limited availability	Variable
Sensory-adapted environment	Reduction of sensory overload (light, sound, tactile stimuli)	Low	Patients with IDD/ASD	Improves comfort in sensitive patients	Requires environmental modification	High
Animal-assisted therapy (AAT)	Emotional regulation and physiological calming	Low	Children	Reduces stress and improves mood	Hygiene, logistics, and certification requirements	High
Er:YAG laser	Reduced vibration, noise, and invasiveness	Moderate	Children	Better acceptance; minimally invasive	High cost; technique-sensitive	High
Audiovisual distraction (VR)	Multisensory distraction competing with pain perception	Moderate	Children	Engaging; reduces anxiety	Content-dependent effectiveness	High

Abbreviations: ASD, autism spectrum disorder; CCLAD, computer-controlled local anesthetic delivery; Er:YAG, erbium-doped yttrium aluminum garnet; VR, virtual reality.

### Types of interventions identified

The interventions included were classified into three main categories:

Technological and physical approaches, e.g., computer-controlled local anesthesia, needle-free delivery systems, Er:YAG lasers, transcutaneous electrical nerve stimulation (TENS), dental operating microscope.

Sensory and relaxation-based strategies, e.g., aromatherapy, hypnosis, breathing exercises, music-based distraction, sensory-adapted environments.

Digital and interactive tools, e.g., mobile applications, humanoid robots, therapy dogs, audiovisual distraction, communication aids.

A synthesis of intervention groups, mechanisms of action, and target populations is presented below.

Effectiveness ratings were based on consistency of findings, study quality, and overall certainty of evidence across included studies.

### Anxiety-reduction outcomes

The majority of included studies reported reductions in anxiety or improvements in patient cooperation.

- Computer-controlled anesthesia systems (such as The Wand or STA) consistently resulted in lower anxiety and pain scores in both children and adults.
- Er:YAG laser therapy reduced auditory and tactile discomfort, improving acceptance in pediatric patients.
- TENS demonstrated anxiolytic effects, particularly in children, attributed to neuromodulation and distraction via the gate control mechanism.
- Sensory-based interventions notably lavender aromatherapy, citrus fragrances, guided breathing, and hypnosis were associated with reductions in physiological and behavioral anxiety indicators.
- Digital technologies, including mobile applications and humanoid robots, enhanced cooperation and reduced anxiety in children by providing engagement, familiarity, and distraction.
- Animal-assisted therapy showed strong emotional regulation benefits, especially in pediatric patients with high baseline fear.

### Certainty of evidence (GRADE)

The certainty of evidence varied across intervention categories. High-certainty evidence was identified for computer-controlled local anesthesia systems, supported by consistent findings from multiple well-designed randomized controlled trials with low risk of bias [19–22, 91]. Moderate-certainty evidence was observed for aromatherapy, Er:YAG laser interventions, and audiovisual distraction techniques [23, 53–63, 97]. Although these approaches demonstrated generally positive effects in reducing dental anxiety, some heterogeneity in study design, intervention protocols, and outcome measures resulted in downgrading for inconsistency and imprecision. Low-certainty evidence was assigned to interventions such as transcutaneous electrical nerve stimulation (TENS), hypnosis, mobile applications, humanoid robots, and animal-assisted therapy [1, 32, 40–44, 67]. These ratings were primarily due to methodological limitations, including small sample sizes, lack of blinding, variability in anxiety assessment tools, and limited replication across studies. Overall, the certainty of evidence was influenced by heterogeneity in study populations (children vs. adults), variability in clinical settings, and differences in outcome measurement instruments. A detailed GRADE assessment, including domain-specific judgments, is presented in Table 3.

**TABLE 3:** GRADE assessment of selected non-pharmacological interventions for dental anxiety (systematic review, global studies, 1971–2024).

Intervention	Study design	Risk of bias	Inconsistency	Indirectness	Imprecision	Publication bias	Certainty of evidence
Computer-controlled anesthesia (CCLAD/STA)	RCTs	Low	Low	Low	Low	Undetected	High
Aromatherapy	RCTs	Moderate	Moderate	Low	Moderate	Possible	Moderate
Breathing exercises	RCTs/crossover studies	Moderate	Moderate	Low	Moderate	Possible	Moderate
Er:YAG laser	RCTs + quasi	Moderate	Moderate	Low	Moderate	Possible	Moderate
TENS	Small RCTs	High	Moderate	Low	High	Possible	Low
Hypnosis	Small RCTs/observational	High	High	Moderate	High	Possible	Low
Mobile applications	RCTs (small)	Moderate	High	Moderate	High	Possible	Low
Humanoid robots	Small RCTs	Moderate	Moderate	Moderate	High	Possible	Low
Audiovisual distraction (VR)	RCTs	Moderate	Moderate	Low	Moderate	Possible	Moderate
Animal-assisted therapy	Observational/RCTs	High	Moderate	Moderate	High	Possible	Low

## Discussion

Dental anxiety remains a major challenge in contemporary dental practice and may negatively influence treatment acceptance, cooperation, appointment attendance, and oral health outcomes [1–6, 90]. The present systematic review evaluated a broad spectrum of non-pharmacological and technology-assisted interventions aimed at reducing anxiety in dental settings. Overall, the included studies demonstrated that many behavioral, sensory, technological, and environmental approaches may contribute to improved patient comfort and reduced procedural stress, although the certainty of evidence varied substantially across intervention categories. Computer-controlled local anesthetic delivery systems (CCLAD) were among the most consistently investigated technological approaches. Several studies demonstrated lower pain perception and reduced anxiety during local anesthesia administration compared with conventional syringes, particularly in pediatric patients. These findings may be associated with slower and more controlled anesthetic delivery, which reduces tissue pressure and injection discomfort [19–22, 91, 96–117]. Additional studies evaluating needle-free anesthesia systems and modified injection techniques also reported encouraging results [98, 102, 118, 119]. Reducing visual exposure to injection devices and minimizing injection discomfort may improve patient acceptance and cooperation during treatment. However, not all studies demonstrated uniformly positive psychological outcomes. Rizzo-Lorenzo et al. [91] reported that detailed information concerning computerized anesthesia systems did not reduce anxiety and, in some patients, increased anticipatory stress. Similar observations suggested that procedural information may either alleviate or intensify anxiety depending on communication style, content framing, and individual coping characteristics. Earlier studies also indicated that psychological stress-reduction strategies may significantly influence emotional responses during dental procedures [88, 120]. TENS and vibratory stimulation techniques were evaluated as adjunctive methods for reducing discomfort associated with local anesthesia administration [23, 24, 32]. Reductions in anxiety and fear were observed among children receiving transcutaneous electrical nerve stimulation, while vibratory stimulation techniques were associated with lower pain perception during local anesthesia administration [32, 34, 35]. These findings are consistent with the gate control theory and related concepts of pain modulation [11, 12]. Electrical stimulation may additionally influence endogenous neuropeptide release associated with pain control mechanisms [121]. Acupressure-based interventions were also investigated in several studies. Reduced need for dental injections during prosthodontic procedures and decreased anxiety levels in pediatric dental patients were reported following acupressure interventions [122, 123]. Although these approaches appear promising, the available evidence remains limited by small sample sizes and methodological heterogeneity. Laser-assisted dentistry represented another important technological category evaluated in the included studies [23, 97, 103–107]. Lower stress and anxiety levels during restorative procedures performed using Er:YAG laser systems compared with conventional rotary instrumentation were reported in several studies [23, 94]. Reduced vibration, noise, and tactile discomfort associated with laser-assisted procedures may contribute to improved patient comfort, particularly in pediatric populations. Nevertheless, despite favorable findings, widespread implementation of laser technologies may still be limited by equipment costs, availability, and the need for specialized operator training [103, 105, 107]. Behavioral and distraction-based interventions were among the most extensively studied approaches identified in this review. Audiovisual distraction, virtual reality systems, mobile applications, and robotic interaction generally demonstrated beneficial effects in reducing anxiety and improving cooperation during dental treatment, especially in children [67, 92, 93, 124–131]. Improved behavior and lower anxiety scores were reported in pediatric patients exposed to audiovisual or interactive distraction techniques [26, 36, 74, 124]. Lower anxiety levels were also observed when dental operating microscope video output was used during restorative treatment [16, 93]. Positive behavioral responses associated with robotic interaction during pediatric dental procedures were also reported. The theoretical basis for distraction techniques involves attentional diversion as an important mechanism in pain coping and anxiety reduction [92, 132]. Earlier reviews also emphasized the beneficial role of distraction techniques in pediatric procedural anxiety management [111, 112]. Mobile applications and digital preparation tools have also gained increasing attention in recent years [15]. Digital preparation applications may improve familiarity with medical and dental procedures before treatment [24, 25]. A mobile application designed for pediatric dental preparation was reported to reduce anxiety more effectively than the tell-show-do technique [26]. Virtual reality and smartphone-based interventions may therefore represent useful adjunctive strategies in pediatric dental anxiety management, although additional high-quality trials remain necessary [15].

Breathing exercises, relaxation techniques, hypnosis, and behavioral desensitization strategies were also investigated in multiple studies [40, 54, 125, 133]. Reductions in anxiety and pain perception following diaphragmatic breathing and bubble-blowing exercises during dental procedures were reported in both pediatric and adult patients [37, 42, 43]. Evidence regarding hypnosis was somewhat more heterogeneous, although several studies demonstrated reductions in procedural anxiety associated with hypnotic interventions and relaxation techniques [39, 40, 96]. Beneficial effects of behavioral rehearsal and video-based desensitization in patients with dental fear were also described [130]. Considerable variability in individual responsiveness to hypnosis may partly explain differences observed between studies [116].

Aromatherapy represented one of the most frequently investigated complementary approaches included in this review [59–63, 126]. Reductions in anxiety levels associated with lavender or orange essential oil exposure were reported in both pediatric and adult dental patients [32, 51, 63, 134, 135]. Systematic reviews further suggested that aromatherapy may provide beneficial anxiolytic effects in dental settings, although the certainty of evidence remains moderate because of heterogeneity in essential oil concentration, duration of exposure, and outcome assessment methods [44, 62]. Lavender and rosemary aromas may additionally influence mood and cognitive processing through neurophysiological mechanisms [51]. Despite generally favorable findings, aromatherapy protocols remain insufficiently standardized across studies. Environmental and interpersonal factors also appeared to influence patients’ emotional responses during dental treatment [29–31, 75–86, 120, 136]. Dentist attire, communication style, and clinic atmosphere may affect children’s cooperation and anxiety perception [76, 77, 79, 84]. Earlier observations additionally indicated that waiting room conditions and waiting times may contribute to elevated anxiety levels before treatment [134]. Sensory-adapted environments were associated with lower stress levels, particularly among children and patients with developmental disorders or sensory sensitivities [30, 31, 78, 86]. Simple modifications involving communication style, clinic atmosphere, and sensory adaptation may therefore be feasible even in routine dental practice and could contribute to improved patient comfort and cooperation. Animal-assisted interventions remain relatively underexplored in dentistry, although preliminary findings appear encouraging [67–74]. Reduced anxiety levels in children treated in the presence of therapy dogs and positive behavioral effects associated with facility dogs in dental care settings have been reported [67, 72]. Animal-assisted activities may additionally improve emotional comfort in pediatric healthcare environments [74]. At the same time, the importance of infection control, allergy prevention, and patient safety when implementing animal-assisted interventions in clinical practice has also been emphasized [73]. Several emerging or less frequently investigated interventions were also identified in the included studies. Reductions in stress and anxiety during peripheral intravenous cannulation associated with green color exposure, as well as possible antinociceptive effects of green light exposure, have been reported in preliminary investigations [135–137]. Although these findings remain preliminary, they may indicate potential directions for future research involving multisensory anxiety-reduction approaches in dentistry. From a clinical perspective, many of the evaluated interventions are relatively inexpensive, easy to implement, and may reduce the need for pharmacological sedation in selected patients. However, substantial heterogeneity between studies, differences in anxiety assessment methods, and variability in intervention protocols limit direct comparison of results. In addition, several included studies involved relatively small sample sizes or short follow-up periods, reducing the overall certainty of evidence. Primary empirical studies were included in the qualitative synthesis, while selected reviews and theoretical publications were used as background references in the Introduction and Discussion. Future research should focus on standardized outcome measures, larger multicenter randomized controlled trials, and long-term evaluation of intervention effectiveness in diverse patient populations. Further investigation is also needed to determine which combinations of behavioral, sensory, and technological interventions provide the greatest benefit in routine clinical dental practice.

### Implications for dental education

The findings of this review may also be relevant for dental education and clinical training. Several studies demonstrated that non-pharmacological strategies, including communication techniques, audiovisual distraction, breathing exercises, sensory adaptation, and behavioral management, may help reduce anxiety and improve patient cooperation during dental treatment [26, 29, 30, 37, 77, 94]. Beneficial effects of distraction-based interventions and breathing exercises were observed in pediatric patients undergoing dental procedures [26, 37]. Environmental and interpersonal factors also appeared important. Dentist behavior, clinic atmosphere, and waiting room adaptations may influence children’s emotional responses and cooperation during treatment [29, 77, 78]. Similarly, sensory-adapted environments were associated with lower stress levels in children, particularly among patients with developmental disorders or sensory sensitivities [30, 31]. These findings suggest that undergraduate and postgraduate dental education could benefit from greater emphasis on behavioral management, patient-centered communication, and recognition of dental anxiety. Greater awareness of non-pharmacological anxiety management strategies may help future clinicians improve patient comfort and treatment acceptance in routine dental practice.

## Conclusion

Dental anxiety remains a significant challenge that may negatively affect treatment acceptance, patient cooperation, and oral health outcomes. This systematic review suggests that several non-pharmacological and physical interventions may help reduce anxiety and improve the dental experience in both pediatric and adult patients.

The strongest evidence supported computer-controlled local anesthetic delivery systems, while moderate-certainty evidence was identified for aromatherapy, audiovisual distraction, breathing exercises, and selected laser-assisted procedures. Many interventions were relatively simple, non-invasive, and feasible for implementation in routine dental practice, particularly those involving communication strategies, sensory adaptation, and behavioral support.

The findings also emphasize the importance of patient-centered communication and individualized anxiety management in contemporary dental care and education. Future studies should focus on standardized methodologies, validated anxiety assessment tools, and high-quality randomized controlled trials to strengthen the evidence base for non-pharmacological dental anxiety management.

## Data Availability

All data generated or analyzed during this study are included in this published article and its Supplementary Material. As this is a systematic review, no new datasets were created.

## Supplementary material

SUPPLEMENTARY MATERIAL 1 Complete database-specific search strategies used for PubMed/MEDLINE, Scopus, Dentistry & Oral Sciences Source, and Google Scholar.

SUPPLEMENTARY MATERIAL 2 Detailed characteristics of the studies included in the qualitative synthesis.
