# Supplementary material for: Non-pharmacological and physical strategies for managing dental anxiety: a systematic review with implications for dental education
Source: Public Health Rev. 2026 Jul 31;47:1609316. doi: 10.3389/phrs.2026.1609316 (PMC13472981; doi:10.3389/phrs.2026.1609316)
Supplement: Supplementary file 1 [file DataSheet1.docx]

Supplementary File 1

**Search Strategy and Study Selection Details**

*Non-pharmacological and physical strategies for managing dental anxiety: A systematic review with implications for dental education*

**Purpose of this supplementary file.** This file provides detailed search strategy, eligibility framework, study selection process, and reasons for exclusion used in the systematic review. It complements the PRISMA 2020 flow diagram and the main Methods section of the manuscript.

# 1. Information sources and search period

| **Item** | **Description** |
| --- | --- |
| **Databases searched** | PubMed/MEDLINE; Scopus; Dentistry & Oral Sciences Source (EBSCO); Google Scholar. |
| **Additional source** | Reference lists of included studies and relevant reviews were screened manually. |
| **Search period** | January 1971 to December 2024. |
| **Languages considered** | English, Polish, and German. |
| **Reference management** | Duplicate records were removed using Mendeley reference management software. |
| **Reporting framework** | The review was conducted according to PRISMA 2020 and prospectively registered in PROSPERO (CRD420251229878). |

# 2. Database-specific search strategies

*The exact syntax was adapted to the indexing structure and search interface of each database. Search terms combined concepts related to dental anxiety and non-pharmacological, physical, sensory, behavioral, and technology-assisted interventions.*

| **Database** | **Search strategy** |
| --- | --- |
| **PubMed/MEDLINE** | (("dental anxiety"[MeSH Terms] OR "dental anxiety" OR "dental fear" OR dentophobia OR odontophobia OR "dental phobia") AND ("non-pharmacological" OR nonpharmacological OR "behavior management" OR "behaviour management" OR "patient cooperation" OR "audiovisual distraction" OR "virtual reality" OR "mobile application" OR "smartphone application" OR aromatherapy OR hypnosis OR relaxation OR "breathing exercise" OR biofeedback OR "transcutaneous electrical nerve stimulation" OR TENS OR "vibratory stimulation" OR acupressure OR "computer-controlled local anesthesia" OR CCLAD OR "single tooth anesthesia" OR STA OR "needle-free anesthesia" OR "jet anesthesia" OR "Er:YAG laser" OR "laser dentistry" OR "sensory adapted dental environment" OR "waiting room" OR "animal-assisted therapy" OR "therapy dog" OR robot OR robotics)) |
| **Scopus** | TITLE-ABS-KEY(("dental anxiety" OR "dental fear" OR dentophobia OR odontophobia OR "dental phobia") AND ("non-pharmacological" OR nonpharmacological OR "behavior management" OR "behaviour management" OR "audiovisual distraction" OR "virtual reality" OR "mobile app*" OR "smartphone app*" OR aromatherapy OR hypnosis OR relaxation OR "breathing exercise*" OR biofeedback OR TENS OR "transcutaneous electrical nerve stimulation" OR "vibratory stimulation" OR acupressure OR "computer-controlled local anesthesia" OR CCLAD OR "single tooth anesthesia" OR STA OR "needle-free anesthesia" OR "jet anesthesia" OR "Er:YAG laser" OR "laser dentistry" OR "sensory adapted dental environment" OR "animal-assisted therapy" OR robot* OR robotics)) |
| **Dentistry & Oral Sciences Source (EBSCO)** | (("dental anxiety" OR "dental fear" OR dentophobia OR odontophobia OR "dental phobia") AND ("non-pharmacological" OR nonpharmacological OR "behavior management" OR "behaviour management" OR "audiovisual distraction" OR "virtual reality" OR "smartphone application" OR "mobile application" OR aromatherapy OR hypnosis OR relaxation OR "breathing exercise" OR biofeedback OR TENS OR "transcutaneous electrical nerve stimulation" OR vibration OR acupressure OR "computer-controlled anesthesia" OR CCLAD OR "single tooth anesthesia" OR "needle-free anesthesia" OR "jet anesthesia" OR "Er:YAG laser" OR "laser dentistry" OR "sensory adapted dental environment" OR "animal-assisted therapy" OR robotics)) |
| **Google Scholar** | Searches were performed using combined keyword strings, including: "dental anxiety" "non-pharmacological" intervention; "dental fear" "virtual reality" children; "dental anxiety" aromatherapy; "dental anxiety" hypnosis; "dental anxiety" TENS; "dental injection" vibration pain anxiety; "computer-controlled anesthesia" dental anxiety; "needle-free anesthesia" dental anxiety; "Er:YAG laser" dental anxiety children; "sensory adapted dental environment" dental anxiety; "therapy dog" dental anxiety; "robot" dental anxiety children. The first relevant pages of results were screened, and reference lists of eligible articles were searched manually. |

# 3. Eligibility criteria

| **PICOS element / criterion** | **Definition used in this review** |
| --- | --- |
| **Population** | Children, adolescents, or adults reporting dental anxiety, dental fear, dentophobia, or anxiety-related responses during dental treatment. |
| **Intervention** | Non-pharmacological, physical, sensory, behavioral, or technology-assisted strategies implemented in a dental setting. |
| **Comparator** | Conventional anxiety-management methods, standard care, placebo, no intervention, or alternative non-pharmacological intervention. |
| **Outcomes** | Changes in anxiety level, treatment cooperation, pain perception, fear scores, behavioral distress, or physiological stress markers such as heart rate, blood pressure, or salivary cortisol. |
| **Study design** | Randomized controlled trials, quasi-experimental studies, controlled clinical studies, crossover studies, split-mouth studies, observational studies, and other primary empirical clinical studies. |
| **Language** | English, Polish, or German. |
| **Publication period** | 1971-2024. |

# 4. Exclusion criteria

**•** Animal studies, in vitro studies, and simulation-based studies without clinical patient data.

**•** Dental interventions unrelated to anxiety, dental fear, cooperation, pain perception, or procedural stress.

**•** Exclusively pharmacological or sedation-only interventions without a non-pharmacological or physical component.

**•** Narrative reviews, editorials, letters, theoretical papers, clinical guidelines, and conference abstracts without original empirical data were not included in the qualitative synthesis; when relevant, selected reviews and theoretical papers were used only as background references.

**•** Duplicate publications, overlapping study reports, or studies with insufficient methodological information.

**•** Articles unavailable as full text after screening, if applicable.

# 5. Study selection process

| **Stage of selection** | **Number of records/reports** |
| --- | --- |
| Records identified through database searching | **832** |
| Duplicate records removed before screening | **214** |
| Records screened by title and abstract | **618** |
| Records excluded after title and abstract screening | **469** |
| Reports sought for retrieval | **149** |
| Reports not retrieved | **0** |
| Full-text reports assessed for eligibility | **149** |
| Full-text reports excluded | **23** |
| Studies included in qualitative synthesis | **126** |

**Screening procedure.** Two reviewers independently screened titles and abstracts. Full texts of potentially eligible reports were then assessed according to the predefined eligibility criteria. Disagreements were resolved by discussion until consensus was reached.

**Reasoning for title/abstract exclusions.** The majority of records excluded during title and abstract screening were unrelated to dental anxiety management, focused exclusively on pharmacological interventions, did not include original clinical data, or did not report anxiety-related outcomes relevant to dental care.

# 6. Reasons for exclusion at full-text eligibility assessment

| **Reason for exclusion** | **n** |
| --- | --- |
| Not related to dental anxiety outcomes | **8** |
| Pharmacological-only interventions | **5** |
| Review articles/editorials without original data | **4** |
| Insufficient methodological information | **3** |
| Duplicate or overlapping publications | **3** |
| **Total full-text reports excluded** | **23** |

# 7. Data extraction variables

| **Variable group** | **Extracted data** |
| --- | --- |
| **Bibliographic data** | First author, year of publication, reference number. |
| **Study context** | Country or region, clinical setting, and patient population where available. |
| **Design and sample** | Study design, number of participants, age group, and participant characteristics. |
| **Intervention details** | Type of non-pharmacological, physical, sensory, behavioral, or technology-assisted intervention. |
| **Comparator** | Conventional care, no intervention, placebo, or alternative comparator. |
| **Outcome measures** | Dental anxiety scales, fear measures, pain scales, cooperation/behavior scores, physiological stress markers, and treatment acceptance. |
| **Main findings** | Direction and clinical interpretation of the reported anxiety-related outcomes. |

# 8. Risk of bias and certainty assessment

| **Assessment component** | **Approach** |
| --- | --- |
| **Randomized controlled trials** | Assessed using the Cochrane Risk of Bias 2 (RoB 2) tool. |
| **Non-randomized and observational studies** | Assessed using Joanna Briggs Institute (JBI) critical appraisal tools. |
| **GRADE domains** | Risk of bias, inconsistency, indirectness, imprecision, and publication bias. |
| **Narrative synthesis** | A narrative synthesis was performed due to heterogeneity in interventions, study populations, and outcome measures. Meta-analysis was not attempted. |

# 9. Relationship with other supplementary material

**Supplementary File 1** documents the search strategy, eligibility criteria, and study selection process. **Supplementary File 2** provides detailed characteristics of studies included in the qualitative synthesis, including study design, population, intervention category, main intervention, and main outcomes.
