# Supplementary material for: Non-pharmacological and physical strategies for managing dental anxiety: a systematic review with implications for dental education
Source: Public Health Rev. 2026 Jul 31;47:1609316. doi: 10.3389/phrs.2026.1609316 (PMC13472981; doi:10.3389/phrs.2026.1609316)
Supplement: Supplementary file 2 [file DataSheet2.docx]

**Supplementary File 2
Characteristics of included studies and selected background references**

This supplementary file summarizes the characteristics of included studies and selected background references used in the systematic review on non-pharmacological and physical strategies for managing dental anxiety. The table includes publication details, study design or source type, population, intervention category, main intervention, and main anxiety-related outcome or relevance to the review.

Supplementary Table S2. Characteristics of included studies and selected background references used in the review, including publication details, study design or source type, population, intervention category, main intervention, and main anxiety-related outcome or relevance to the review.

| **Ref. No.** | **Author (Year)** | **Study Design** | **Population** | **Intervention Category** | **Main Intervention** | **Main Outcome** |
| --- | --- | --- | --- | --- | --- | --- |
| 1 | Cebalo et al. (2020) | Clinical study | Children (9–14 years) | TENS / neuromodulation | Transcutaneous electrical nerve stimulation | Reduced anxiety and fear |
| 2 | Suresh et al. (2020) | Observational study | Preschool children | Behavioral management | Doll placement test | Behavioral assessment during first dental visit |
| 3 | Gómez-Polo et al. (2021) | Randomized clinical trial | Pediatric patients | Virtual reality | VR distraction | Reduced anxiety and improved cooperation |
| 4 | Ahmed et al. (2016) | Review article | Dental patients | Anxiety management | Psychological management strategies | Improved understanding of dental anxiety |
| 5 | Cunningham et al. (2021) | Systematic review | Pediatric patients | Digital interventions | VR and smartphone applications | Reduction of pediatric dental anxiety |
| 6 | Ghaderi & Solhjou (2020) | Randomized clinical trial | Children | Aromatherapy | Lavender aromatherapy | Reduced stress and pain perception |
| 7 | Arrow & Klobas (2017) | Randomized controlled trial | Children | Minimal intervention dentistry | Non-invasive restorative care | Reduced dental anxiety |
| 8 | Tan (2021) | Review article | Dental patients | Local anesthesia | Comfort-enhancing anesthesia techniques | Reduced discomfort during injections |
| 9 | Heaton et al. (2013) | Randomized clinical trial | Adults | Computer-controlled anesthesia | Injection fear treatment | Reduced dental injection fear |
| 10 | Rizzo-Lorenzo et al. (2020) | Randomized controlled trial | Adults | Computer-controlled anesthesia | Information about computerized anesthesia | Increased anticipatory anxiety |
| 11 | Naini et al. (1999) | Review article | Dental patients | Fear management | Psychological/pharmacological approaches | Anxiety reduction strategies |
| 12 | Kour et al. (2017) | Clinical comparative study | Children | Injection modification | Insulin syringe | Reduced anxiety during anesthesia |
| 13 | Ghasemi et al. (2014) | Comparative clinical study | Children | Injection technique | 30G vs 27G needles | Differences in pain perception |
| 14 | Prabhu et al. (2013) | Randomized clinical trial | Adults | Injection modification | Insulin syringe | Reduced injection pain |
| 15 | Shankar et al. (2022) | Split-mouth randomized trial | Adults | Needle-free anesthesia | Jet anesthesia | Improved patient comfort |
| 16 | Steenberghe et al. (2004) | Multicenter crossover study | Adults | Topical anesthesia | Non-injectable anesthetic gel | Improved comfort during periodontal treatment |
| 17 | Gozdemir et al. (2016) | Randomized controlled trial | Adults | Needle-free anesthesia | INJEX™ system | Reduced needle-related discomfort |
| 18 | Oliveira et al. (2019) | Randomized clinical trial | Adults | Needle-free anesthesia | Jet injection anesthesia | Reduced pain perception |
| 19 | Melwani et al. (2018) | Comparative clinical study | Children | Behavioral modification | Camouflaged syringes | Reduced anxiety and improved behavior |
| 20 | Nanitsos et al. (2009) | Clinical trial | Adults | Vibratory stimulation | Vibration device during injection | Reduced injection pain |
| 21 | Ghorbanzadeh et al. (2019) | Clinical trial | Adults | Vibratory stimulation | Vibratory stimulation during local anesthesia | Reduced pain perception |
| 22 | Taymour et al. (2019) | Clinical study | Adults | Acupressure | Acupressure during prosthodontics | Reduced need for anesthesia |
| 23 | Avisa et al. (2018) | Clinical study | Children | Acupressure | Acupressure therapy | Reduced dental anxiety |
| 24 | Campanella et al. (2018) | Cross-over study | Adults | Single-tooth anesthesia | STA system | Reduced pain and anxiety |
| 25 | Giannetti et al. (2018) | Clinical experimental study | Pediatric patients | Single-tooth anesthesia | STA system | Improved patient acceptance |
| 26 | Aggarwal et al. (2018) | Comparative clinical study | Adults | Computer-controlled anesthesia | CCLAD system | Lower anxiety and pain |
| 27 | Patini et al. (2018) | Randomized clinical trial | Children | Computer-controlled anesthesia | Computer-controlled delivery system | Reduced heart rate and pain |
| 28 | Shindova et al. (2018) | Clinical study | Children | Laser-assisted dentistry | Er:YAG laser | Reduced stress during sealant application |
| 29 | Sayed et al. (2016) | Clinical study | Children | Audiovisual distraction | Dental operating microscope video output | Reduced anxiety |
| 30 | Kasimoglu et al. (2020) | Clinical trial | Children | Robotics | Humanoid robot interaction | Reduced dental anxiety |
| 31 | Al-Khotani et al. (2016) | Randomized clinical trial | Children | Audiovisual distraction | Audiovisual distraction | Improved behavior during treatment |
| 32 | El-Sharkawi et al. (2012) | Clinical study | Children | Distraction technique | Audiovisual distraction | Reduced pain during injections |
| 33 | Elicherla et al. (2019) | Randomized controlled trial | Children | Mobile applications | “Little Lovely Dentist” app | Reduced dental anxiety |
| 34 | Patil et al. (2017) | Pilot study | Children | Dental applications | Dental behavior management apps | Improved behavior |
| 35 | Shah et al. (2017) | Comparative clinical study | Pediatric patients | Smartphone applications | Dental apps vs traditional techniques | Reduced anxiety and physiological stress |
| 36 | Levi et al. (2022) | Feasibility crossover study | Children | Breathing techniques | Diaphragmatic breathing | Reduced dental fear |
| 37 | Peretz & Gluck (1999) | Clinical study | Children | Active distraction | Deep breathing and blowing air | Reduced injection anxiety |
| 38 | Moore et al. (1996) | Comparative study | Adults | Hypnosis | Group therapy vs hypnosis | Reduced dental anxiety |
| 39 | Mc Ammond et al. (1971) | Experimental study | Adults | Hypnosis and relaxation | Hypnosis training | Reduced stress reactions |
| 40 | Katcher et al. (1984) | Clinical study | Adults | Hypnosis | Contemplation and hypnosis | Reduced surgical anxiety |
| 41 | Bahrololoomi et al. (2022) | Crossover RCT | Children | Breathing exercise | Bubble blower breathing | Reduced anxiety and pain |
| 42 | Morarend et al. (2011) | Exploratory study | Adults | Biofeedback | Respiratory rate biofeedback | Reduced dental anxiety |
| 43 | Zabirunnisa et al. (2014) | Clinical study | Adults | Aromatherapy | Lavender fragrance | Reduced patient anxiety |
| 44 | Arslan et al. (2020) | Randomized clinical trial | Children | Aromatherapy | Lavender oil inhalation | Reduced anxiety and pain |
| 45 | Jafarzadeh et al. (2013) | Randomized clinical trial | Children | Aromatherapy | Orange essential oil | Reduced cortisol and pulse rate |
| 46 | Lehrner et al. (2000) | Clinical study | Adults | Aromatherapy | Orange ambient odor | Reduced anxiety and improved mood |
| 47 | Kritsidima et al. (2010) | Cluster randomized trial | Adults | Aromatherapy | Lavender scent | Reduced anxiety levels |
| 48 | Toet et al. (2010) | Comparative study | Adults | Ambient fragrance | Pleasant environmental odors | Reduced dental fear |
| 49 | Welly et al. (2012) | Psychophysiological study | Children | Dental atmosphere | Dentist behavior and atmosphere | Improved cooperation |
| 50 | Yahyaoglu et al. (2018) | Clinical study | Children | Dentist appearance | Dentist attire | Influence on dental fear |
| 51 | Potter et al. (2019) | Preliminary study | Individuals with disabilities | Sensory adaptation | Sensory-adapted routine dental care | Reduced anxiety |
| 52 | Sujatha et al. (2021) | Cross-sectional study | Children | Dentist attire | Child preference for attire | Reduced anxiety |
| 53 | Fux-Noy et al. (2019) | Case-control study | Children | Waiting room adaptation | Modified waiting room environment | Reduced anxiety |
| 54 | Shapiro et al. (2007) | Clinical study | Children | Sensory adaptation | Adapted dental environment | Reduced physiological stress |
| 55 | Cermak et al. (2015) | Pilot randomized study | Children with ASD | Sensory-adapted environment | Adapted sensory environment | Improved cooperation |
| 56 | Thakkar et al. (2021) | Randomized controlled trial | Children | Animal-assisted therapy | Therapy dog | Reduced dental anxiety |
| 57 | Gussgard et al. (2019) | Clinical review | Dental patients | Dog-assisted therapy | Therapy dog implementation | Risk assessment |
| 58 | Glaesmer et al. (2015) | Controlled clinical trial | Adults | Hypnosis | Hypnosis before tooth removal | Reduced anxiety |
| 59 | Takemura et al. (2021) | Clinical study | Adults | Environmental modification | Green color exposure | Reduced stress and pain |
| 60 | Vishwakarma et al. (2017) | Randomized controlled trial | Children | Behavioral modification | Behavioral management techniques | Reduced anxiety |
| 61 | Moore et al. (1991) | Clinical comparative study | Adults | Behavioral therapy | Video training and clinical rehearsal | Reduced dental fear |
| 62 | Corah et al. (1979) | Clinical study | Adults | Stress reduction | Psychological stress reduction techniques | Reduced procedural stress |
| 63 | Reed et al. (2012) | Review article | Dental patients | Local anesthesia | Technical anesthesia considerations | Improved injection comfort |
| 64 | Angelo & Polyvios (2018) | Review article | Dental patients | Alternative anesthesia | Alternative anesthesia techniques | Reduced discomfort |
| 65 | Second & Neelakantan (2014) | Review article | Dental patients | Local anesthesia | New anesthesia delivery methods | Improved patient comfort |
| 66 | Daneswari & Nandlal (2011) | Review article | Children | Laser dentistry | Hard tissue laser use | Reduced anxiety and discomfort |
| 67 | Borsatto et al. (2004) | Experimental clinical study | Children | Laser-assisted dentistry | Er:YAG laser fissure treatment | Improved treatment tolerance |
| 68 | Caprioglio et al. (2017) | Narrative review | Pediatric patients | Laser dentistry | Pediatric laser applications | Improved patient acceptance |
| 69 | Frentzen et al. (2003) | Clinical study | Dental patients | Laser-assisted dentistry | Er:YAG laser fissure sealing | Reduced discomfort |
| 70 | Ibaraki et al. (2003) | Experimental clinical study | Dental patients | Laser-assisted dentistry | Er:YAG laser treatment | Reduced invasive sensation |
| 71 | Parker (2007) | Review article | Dental patients | Surgical lasers | Hard tissue laser applications | Reduced operative discomfort |
| 72 | Beran et al. (2015) | Exploratory study | Children | Robotics | Humanoid robotic interaction | Positive emotional responses |
| 73 | DeMore & Cohen (2005) | Critical review | Children | Distraction therapy | Pediatric distraction techniques | Reduced procedural pain |
| 74 | Slifer et al. (2002) | Review article | Children | Coping strategies | Repeated invasive procedures | Improved coping |
| 75 | Azeta et al. (2018) | Review article | Healthcare patients | Robotics | Humanoid robotics in healthcare | Anxiety reduction potential |
| 76 | Beran et al. (2013) | Clinical trial | Children | Robotics | Humanoid robot during vaccination | Reduced distress |
| 77 | Gherman et al. (2016) | Systematic analysis | Medical patients | Mobile applications | Procedural preparation apps | Improved preparation and reduced anxiety |
| 78 | Zink et al. (2018) | Clinical study | Children with ASD | Communication technology | Communication application | Improved communication and reduced stress |
| 79 | Levi et al. (2022) | Feasibility crossover study | Children | Breathing techniques | Diaphragmatic breathing | Reduced dental fear |
| 80 | Morarend et al. (2011) | Exploratory investigation | Adults | Biofeedback | Respiratory biofeedback device | Reduced dental anxiety |
| 81 | Purohit et al. (2021) | Systematic review and meta-analysis | Dental patients | Aromatherapy | Aromatherapy interventions | Reduced dental anxiety |
| 82 | Jimson et al. (2016) | Review article | Dental patients | Aromatherapy | Aromatherapy in dentistry | Anxiety reduction potential |
| 83 | Lu et al. (1995) | Clinical report | Drug-dependent patients | Hypnosis | Hypnosis-assisted sedation | Improved sedation outcomes |
| 84 | Rodolfa et al. (1990) | Review article | Dental patients | Dental phobia management | Etiology and treatment approaches | Reduced anxiety |
| 85 | Morse & Cohen (1983) | Case report | Adults | Meditation-hypnosis | Needle phobia management | Reduced needle fear |
| 86 | Chouhan et al. (2017) | Review article | General population | Essential oils | Antimicrobial essential oils | Therapeutic potential |
| 87 | Moss et al. (2003) | Experimental study | Healthy adults | Aromatherapy | Rosemary and lavender oils | Mood and cognition effects |
| 88 | Pradopo et al. (2017) | Clinical study | Pediatric patients | Aromatherapy and music | Pandan aromatherapy with relaxation music | Reduced dental anxiety |
| 89 | Fayazi et al. (2011) | Randomized clinical trial | Surgical patients | Aromatherapy | Inhalation aromatherapy | Reduced preoperative anxiety |
| 90 | Conrad & Adams (2012) | Pilot study | Postpartum women | Aromatherapy | Clinical aromatherapy | Reduced anxiety and depression |
| 91 | Wotman et al. (2017) | Clinical study | Surgical patients | Aromatherapy | Lavender aromatherapy | Reduced preoperative anxiety |
| 92 | Ndao et al. (2012) | Placebo-controlled trial | Children and adolescents | Aromatherapy | Inhalation aromatherapy | Improved emotional comfort |
| 93 | Seyyed-Rasooli et al. (2016) | Randomized clinical trial | Burn patients | Aromatherapy massage | Massage and inhalation aromatherapy | Reduced anxiety and pain |
| 94 | Lee et al. (2012) | Overview of systematic reviews | Healthcare patients | Aromatherapy | Aromatherapy interventions | Evidence synthesis |
| 95 | Cai et al. (2021) | Systematic review | Dental patients | Aromatherapy | Aromatherapy efficacy | Reduced dental anxiety |
| 96 | Jadhav & Mittal (2020) | Randomized clinical trial | Adults | Aromatherapy | Aromatherapy during endodontics | Improved anesthesia success |
| 97 | Hosey (2002) | Clinical guideline | Pediatric patients | Conscious sedation | Sedation management | Anxiety management guidance |
| 98 | Walsh (2007) | Review article | Dental patients | Anxiety prevention | 4S principle | Anxiety reduction strategies |
| 99 | Kleinknecht et al. (1973) | Observational study | Adults | Dental fear | Origins of dental fear | Fear-related factors |
| 100 | Klingberg et al. (1995) | Clinical study | Children | Child dental fear | Fear-related factors | Clinical effects of anxiety |
| 101 | Townend et al. (2000) | Clinical study | Children | Dental anxiety | Clinical anxiety assessment | Anxiety-related behaviors |
| 102 | Vagnoli et al. (2005) | Randomized prospective study | Children | Clown therapy | Clown doctors | Reduced preoperative anxiety |
| 103 | Umamaheshwari et al. (2013) | Clinical study | Children | Environmental modification | Child-friendly colors | Reduced anxiety |
| 104 | Babaji et al. (2017) | Clinical study | Children | Dentist attire and syringe camouflage | Behavioral modification | Reduced anxiety |
| 105 | Ikusaka et al. (1999) | Observational study | Adults | Physician attire | No white coat consultations | Patient attitude assessment |
| 106 | Kuscu et al. (2009) | Cross-sectional study | School children | Dentist attire | Attire preferences | Relationship with dental anxiety |
| 107 | Ravikumar et al. (2016) | Observational study | Children | Dentist attire | Pediatric dentist appearance | Child perceptions |
| 108 | Peretz & Efrat (2000) | Clinical study | Adolescents | Dental anxiety | Anxiety prevalence | Anxiety-related findings |
| 109 | Coffey & Di Giusto (1983) | Clinical study | Adults | Waiting room environment | Waiting time and atmosphere | Anxiety reduction |
| 110 | Merrick et al. (2004) | Clinical review | Patients with disabilities | Multisensory stimulation | Snoezelen therapy | Reduced stress |
| 111 | Shapiro et al. (2009) | Clinical study | Children with developmental disabilities | Adapted environment | Sensory adaptation | Reduced anxiety |
| 112 | Kheir et al. (2019) | Cross-sectional study | Young adults | Patient-dentist relationship | Communication and trust | Reduced anxiety |
| 113 | Murad et al. (2020) | Systematic review | Dental patients | Anxiety factors | Fear-associated variables | Anxiety-related predictors |
| 114 | Eggiman (2006) | Case report | Adults | Animal-assisted therapy | Cognitive-behavioral therapy with animals | Anxiety reduction |
| 115 | Ernst (2014) | Review article | Long-term care patients | Animal-assisted therapy | Therapy animal programs | Psychological benefits |
| 116 | Kruger et al. (2004) | Conference report | Adolescents | Animal-assisted intervention | Mental health interventions | Emotional support |
| 117 | Kucera (2021) | Clinical perspective | Dental patients | Facility dogs | Animal-assisted intervention | Reduced dental anxiety |
| 118 | Caprilli & Messeri (2006) | Pilot study | Hospitalized children | Animal-assisted activity | Therapy animals | Reduced anxiety |
| 119 | Flammer & Bongartz (2003) | Meta-analysis | Adults | Hypnosis | Efficacy of hypnosis | Anxiety reduction |
| 120 | Lim & Borromeo (2017) | Review article | Adults with special needs | General anesthesia | Dental treatment under GA | Anxiety management |
| 121 | Lehrner et al. (2005) | Clinical study | Adults | Aromatherapy | Orange and lavender odors | Reduced anxiety and improved mood |
| 122 | Maybodi et al. (2018) | Clinical study | Adults | Aromatherapy and music | Lavender aroma and music | Reduced periodontal surgery anxiety |
| 123 | Venkataramana et al. (2016) | Randomized controlled trial | Adults | Aromatherapy | Aromatherapy during dental treatment | Reduced anxiety |
| 124 | Vishwakarma et al. (2017) | Randomized controlled trial | Children | Behavioral techniques | Behavioral modification methods | Reduced anxiety |
| 125 | Adami et al. (2021) | Clinical trial | Adults | Needle-free anesthesia | Mucoadhesive anesthetic patch | Reduced pain |
| 126 | Theocharidou et al. (2021) | Split-mouth randomized study | Children | Jet anesthesia | Jet vs conventional anesthesia | Reduced discomfort and anxiety |

Abbreviations: ASD – autism spectrum disorder; CCLAD – computer-controlled local anesthetic delivery; Er:YAG – erbium-doped yttrium aluminum garnet; GA – general anesthesia; RCT – randomized controlled trial; STA – single-tooth anesthesia; TENS – transcutaneous electrical nerve stimulation; VR – virtual reality.
